# Virus-like particles (VLP) in prophylaxis and immunotherapy of allergic diseases

**DOI:** 10.1007/s40629-018-0074-y

**Published:** 2018-07-09

**Authors:** Ludger Klimek, Thomas Kündig, Matthias F. Kramer, Sonja Guethoff, Erika Jensen-Jarolim, Carsten B. Schmidt-Weber, Oskar Palomares, Mona O. Mohsen, Thilo Jakob, Martin Bachmann

**Affiliations:** 1 Center for Rhinology & Allergology, Wiesbaden, Germany; 2 Department for Dermatology, University Hospital Zürich, Zurich, Switzerland; 3 Bencard Allergie GmbH, Munich, Germany; 4 Allergy Therapeutics plc, Worthing, UK; 5 Institute for Pathophysiology and Allergy Research, Center for Pathophysiology, Infectiology and Immunology, Medical University of Vienna, Vienna, Austria; 6 Inter-University Messerli Science Institute, Veterinary University Vienna, Vienna, Austria; 7 Center for Allergy and Environmental Resarch (ZAUM), Technical University and Helmholtz-Center, Munich, Germany; 8 Department of Biochemistry and Molecular Biology, School of Chemistry, Complutense University of Madrid, Madrid, Spain; 9 Jenner Institute, University of Oxford, Oxford, UK; 10 Department of Dermatology and Allergology, University Medical Center Gießen and Marburg, Campus Gießen, Justus-Liebig-University, Gießen, Germany; 11 Inselspital, University Department for Rheumatology, Immunology and Allergology, Sahlihaus 1, 3010 Bern, Switzerland

**Keywords:** Adjuvants, Allergic rhinitis, Allergy, CpG motifs, Immunotherapy, Vaccines, Virus-like particles

## Abstract

**Background:**

Apart from active allergen avoidance, immunotherapy is regarded as the most effective form of treatment available for type I allergies. Such treatments involve the administration of allergen preparations in various forms and by various routes. Virus-like particles (VLPs) offer a very effective platform for immunization with the allergen and are characterized by high immunogenicity, low allergenicity and high clinical efficacy. Formulations that include Toll-like receptor ligands, T cell stimulatory epitopes and/or depot-forming adjuvants appear to enhance activation of the relevant immune cells. Short nucleotide sequences including CpG motifs have also been intensively explored as potent stimulators of dendritic cells and B cells.

**Methods:**

The present paper is based on a systematic literature search in PubMed and MEDLINE, and focuses on the pertinent immunological processes and on clinical data relating to use of VLPs and CpG motifs for the treatment of allergic rhinitis (AR).

**Results:**

Many published studies have reported positive clinical results following administration of VLPs, either alone or in combination with CpG motifs and, in some cases, even in the absence of the allergen-specific allergen.

**Conclusions:**

These results indicate that VLPs modulate immune responses in ways which underline their exceptional promise as a platform for the immunotherapy of allergic disorders. However, clinical evaluations remain limited, and further large-scale and longer-term studies will be necessary to substantiate the efficacy and safety of these novel therapies.

## Introduction

There has been a significant increase in the incidence of allergic disorders of the upper respiratory tract in recent decades, and these syndromes are now among the most common of all chronic illnesses in Europe [1–3]. According to the definition proposed by the ARIA (Allergic Rhinitis and its Impact on Asthma) Initiative, allergic rhinitis is an IgE-mediated inflammatory reaction of the nasal epithelium with the corresponding symptoms, which is precipitated by exposure to an allergen [2]. Other than allergen avoidance, the only recognized targeted treatment available for allergic rhinitis is allergen-specific immunotherapy (SIT) [4–7].

SIT generally involves the repeated sublingual or subcutaneous application of an allergen extract in progressively increasing doses during the initial phase, followed by the regular administration of a sustaining dose, if necessary [4–7]. This regimen leads to a reduction in the severity of symptoms and the use of symptomatic medication, enhancement of general wellbeing and further improvements in the patient’s situation [4–7]. In addition, clinical studies have revealed long-term effects that persist beyond the termination of the treatment phase [8, 9]. In the case of subcutaneous immunotherapy (SCIT), it has been demonstrated that the treatment also helps to prevent the re-emergence of sensitization, and progression to bronchial asthma [10, 11]. Among the shortcomings of SIT are the incidence of undesirable side-effects [12], the considerable duration of the treatment (which can cause problems in ensuring adherence to the therapeutic regimen), and the lack of data relating to its efficacy in patients who present with polyvalent sensitization [13]. Various novel approaches have been proposed and debated, which promise treatment regimens that are more effective, less onerous, and of shorter duration [14, 15]. Of these alternative strategies, the most advanced are treatments that employ recombinant (native versions) of major allergens as well as hypoallergenic variants [16], peptide immunotherapy [17], intralymphatic immunotherapy [18], and epicutaneous immunotherapy [19].

Since its inception, allergen-specific immunotherapy has developed in parallel with vaccination. In the early years of the 20th century, the effect of SIT was explicitly equated with that of vaccination against the toxins postulated to be present in pollen. Thus, Dunbar (1903) developed a hyperimmune serum in animals that had been vaccinated with pollen extracts and used this preparation (“Pollantin”), administered as nasal drops, to treat patients suffering from hay fever. Although these efforts might appear rather outlandish to us today, they were in fact well thought out and can be said to have anticipated the concept of blocking antibodies by over a century. Indeed, the analogy between SIT and vaccination has been revived in recent years [20]. Not for nothing the WHO refers to SIT as “allergy vaccination” [4]. Against this background, it seems reasonable to translate other concepts developed in the context of active immunization, such as that of VLPs, into the realm of SIT.

Virus-like particles (VLPs) are of particular interest as a possible basis for the development of an immunotherapy for the treatment of inhalation allergies [21–24].

Pathogen-specific VLP-based vaccines have been shown to be well tolerated and immunologically effective for prophylactic vaccination against papillomavirus and hepatitis B virus and, most recently, against malaria, and are now in use in clinical practice [25–27].

VLPs based on the coat protein of the bacteriophage Qbeta have been used as carriers of diverse target structures to raise antibodies directed against tumors and infectious agents [28]. Moreover, VLP libraries displaying mimotope peptides can serve as an inexhaustible source anti-tumor vaccines [29].

The recent review by Mohsen et al. provides an excellent summary of the rationale for the use of VLPs as antigen carriers in vaccines, discusses the optimal structural characteristics of VLPs as a platform to achieve the desired immunological effects, and provides an overview of the agonists of the Toll-like receptor family (TLT) members, which can be included in VLPs to enhance the immune response [27].

**Fig. 1:**
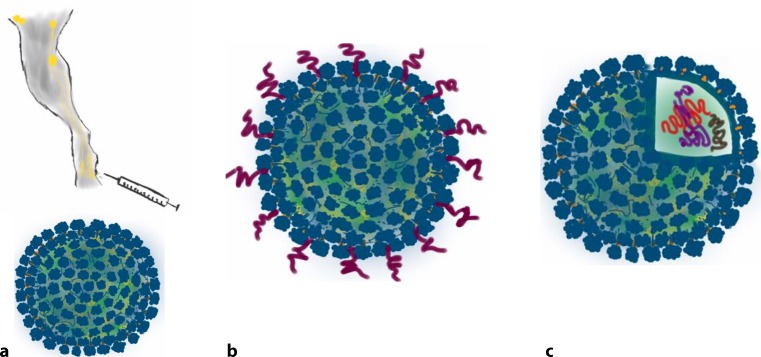
Their size and the ability to modify both their outer surfaces and their contents are the keys to the versatility of VLPs as a vaccine platform. **a** Size and kinetics of VLPs, **b** Modifying the surface of VLPs, **c** Exploiting the interior of VLPs. *VLP* virus-like particle

Fig. 1 summarizes the advantages provided by the VLP platform. With diameters in the 30-nm range, the size of the VLPs is crucial because it allows for their direct drainage into the lymph nodes, which permits optimal B cell activation. Furthermore, the VLP surface can be modified to display the desired epitopes, while the interior of VLPs can be loaded with diverse TLR ligands, such as RNA or DNA sequences enriched in non-methylated CG motifs, which activate TLR7/8 and TLR9, respectively.

**Fig. 2:**
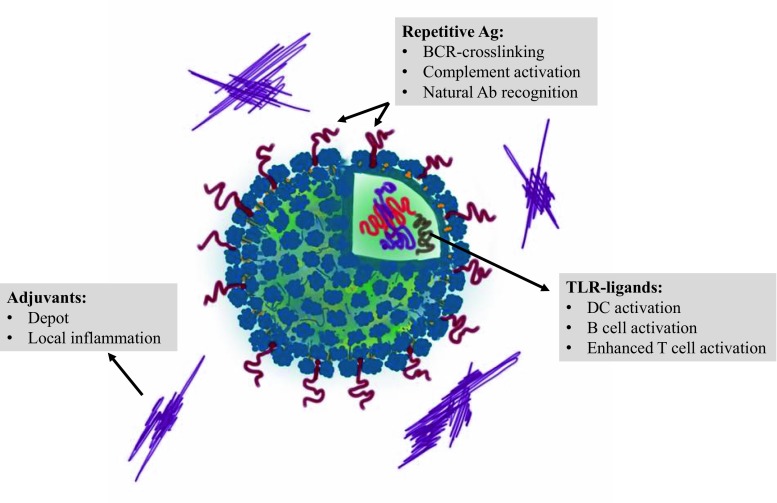
Immunological mechanisms of VLP-based vaccines. *Ab* antibodies; *BCR* B cell receptor; *DC* dendritic cell; *TLR* Toll-like receptor; *VLP* virus-like particle

Bacteriophage-based VLPs—packed with oligonucleotides enriched in CpG motifs (CpGs)—and displaying tumor antigens on their surfaces, have been shown to activate specific T cells in melanoma patients [30]. Similar CpGs have been successfully used as adjuvants in prophylactic vaccinations [31]. In combination with, for instance, CpG motifs, VLP technology could make it possible to effectively treat complex conditions such as malignancies, autoimmune diseases, and allergies at the causative level [22]. Fig. 2 shows how one can optimize the immunogenicity of antigens by presenting them on the surface of the VLPs, loading the VLPs with TLR ligands, and including an adjuvant in the final formulation.

In this article, we therefore review clinical data on the use of VLPs and CpG motifs in the treatment of allergic rhinitis.

## Physiological immune response versus allergic response

The misregulation of the immune response to environmental antigens that triggers the development of allergies is characterized by the excessive (relative to the Th1-type) development of a Th2-type pattern of cytokines dominated by interleukins (IL)-4, -5, and -13, which results in the generation and activation of IgE-producing B lymphocytes and effector cells that generate an allergic inflammatory reaction [33]. IL-17-producing Th17 cells also play a role in provoking the resulting chronic inflammatory reaction. These cells develop as a result of the activation of epithelial cells by transforming growth factor (TGF)-β and IL-6, and are also capable of activating allergic effector cells.

The regulatory cytokines IL-10 and TGF-β suppress both the proliferation of effector T cells and the production of cytokines [33]. They are secreted by a class of T cells that develops from naive precursors, giving rise to an autonomous population of regulatory T cells [32] which are referred to as Treg cells. Tregs are allergen-specific T cells, which are characterized by weak proliferation and the production of immunoregulatory cytokines. In order to execute their regulatory function they also require frequent contact with and activation by the cognate antigen [32], which is mediated by antigen-presenting dendritic cells (DCs). The early stage of Treg development is marked by the expression of the transcription factor FOXP3 (Forkhead box protein 3) [33].

The normal immune response differs from its allergic counterpart in the relatively high numbers of allergen-specific Tregs with regard to the Th1 and Th2 cells [34]. The production of sufficiently large numbers of Treg or Th17 cells relative to effector T cells is necessary to ensure that the immune response is balanced [32]. This requires that all T cell subpopulations be derived from the same naive precursor cells in a process that is controlled by the operation of diverse cytokines and transcription factors [33]. Activated Th1 cells express the transcription factor T‑bet and secrete IL-12, Th2 cells express transcription factor GATA-3 and secrete IL-4 and, while Tregs express the transcription factor FOXP3 and secrete TGF-β [34].

The corresponding transcription factors therefore serve as reliable markers for each of the various T cell subpopulations [32]. It is generally assumed that the vaccination process triggers the development of new T cell populations and that the maturation of these subpopulations is controlled by competition between the transcription factors mentioned above. Ultimately, one or another of these becomes predominant in any one cell, which leads to a polarization of the T cell populations and thus to the functional polarization of the immune response. It is known, for instance, that T‑bet (the Th1-specific transcription factor) and GATA3 (the pro-allergic Th2 transcription factor) are mutually antagonistic. Interestingly, GATA-3 also directly inhibits the expression of the FOXP3 gene [35].

It is therefore conceivable that IL-4 reduces tolerance to antigens generally, and conversely that the counteracting Th1 or Treg population acts to promote tolerance to allergens [36].

These immunological mechanisms also account for the actions of VLPs and CpG motifs in alleviating allergic disorders.

Non-allergic immune responses can be induced either by the use of high concentrations of the appropriate antigen or another immunological trigger, such as an adjuvant or VLPs. For the induction of T cell responses, only the antigen’s binding site for the major histocompatibility complex (MHC) II is essential. Therefore, activation of immunity and tolerance by SIT at the level of the T cell could in principle be achieved using a mixture of peptides bearing T cell-specific epitopes [34]. Allergy sufferers and non-allergic individuals both recognize essentially the same immunogenic determinants (epitopes) in a potential allergen [33]. This is also true of antibodies; whether induced in allergic or non-allergic individuals, they recognize the same, in this case native, epitopes present on allergens.

As with conventional vaccinations, the efficacy of the intervention is strongly dependent on the ability of the inducing agent to trigger a potent and long-lasting induction of antibody production and T‑helper cells, immunological memory cells and a potent B cell response. This is where VLPs offer a potential advantage as immunogens in allergy vaccines [37, 38].

Virus-like particles (VLPs) are nanoparticles that contain no viral RNA or DNA and are therefore incapable of replication. As a rule, VLPs are made up of proteins derived from capsids or coat proteins of either viruses or bacteriophages [21, 22, 27]. VLP-based vaccines against hepatitis B and human papilloma virus and more recently malaria have been shown to be well tolerated, immunogenic, and efficacious for prophylactic vaccination against infectious agents [21, 22, 31]. In addition, VLPs based on the viral coat of bacteriophage Q‑beta (Qb-VLPs) have been used in both preclinical and clinical settings as vehicles for the presentation of antigens in various vaccines directed against neoplasias, as well as disorders characterized by chronic inflammation [21].

Qb-VLPs are each made up of 180 identical copies of the capsid protein of the bacteriophage, and are of the same size and form as the native bacterial virus. VLPs spontaneously incorporate bacterial RNA during their assembly. The protein coat stabilizes the nucleic acids and protects them from enzymatic degradation. Moreover, these Qb-VLPs can be disassembled and reassembled in the presence of oligodeoxynucleotides (ODNs).

## Immune responses induced by VLPs and CpG motifs

The uptake of antigens by antigen-presenting cells (APCs) is strongly dependent on features such as the size, form, surface structure, hydrophobicity, hydrophilicity, and charge distribution, which all contribute to optimal interaction with the MHC-II receptor [27, 39]. The properties of nanoparticles like VLPs are particularly favorable for the promotion of uptake by APCs [40–42]. Once inside the APC the antigen is processed, i.e., degraded into peptides, and these peptides are presented in association with MHC Class I and Class II molecules on the APC surface. Recognition by MHC-II leads to the stimulation of CD4^+^ T cells. Moreover, the size of VLPs, which is of the order of 30 nm, also permits their antigenic peptides to be presented by MHC Class I molecules, which leads to the induction of a CD8^+^ T cell response [43, 44].

Many APCs are dendritic cells (DCs), which can in turn be divided into several populations. Myeloid DCs (mDCs) mainly secrete IL-12, plasmacytoid DCs (pDCs) produce IFN-α, and CD8+ lymphoid DCs can induce the production of cytotoxic T lymphocytes [45–47]. TLR ligands, such as CpG motifs, in turn stimulate the activation of antigen-presenting DCs by binding to the TLRs expressed on the surface of the latter [47, 48].

## CpG motifs

Particular molecular structures found on the surfaces or in internal components of viruses and other microorganisms are among the most potent activators of immune cells in humans. Among these structures are unmethylated CpG oligodinucleotide motifs, which are commonly found in bacterial genomes but are relatively rare in the human genome, in which most CpG dinucleotides are methylated [37, 38].

CpG oligodeoxynucleotides (CpG-ODN) constitute a class of synthetically produced single-stranded oligonucleotides, which contain a relatively high proportion of CG motifs. CG sequence motifs (where C represents the nucleotide cytosine, and G stands for guanine) rarely occur in the human genome: CG accounts for less than 2% of all dinucleotides in the human genome, and in many cases, the C in this context occurs in methylated form [21].

In the genomes of bacteria and viruses, on the other hand, the CG dinucleotide is not only far more common, the cytosine is also predominantly found in the unmethylated state. In humans, the adaptive immune system recognizes unmethylated CG motifs in bacterial and viral nucleic acids by means of TLR9 [21, 38]. Since extracellular DNA is rapidly degraded by enzymes, the nucleotides in the GC-rich bacterial DNA fragments used as an adjuvant with VLPs are usually linked by phosphorothioate groups instead of the phosphodiester bond characteristic of natural DNA. CpG is the conventional abbreviation for the natural dideoxynucleotide. The various types of CpG motifs that have been deployed for SIT are described below.

CpG-ODNs have been used together with tumor antigens to stimulate the induction of specific CD8^+^ and CD4^+^ T cells in melanoma patients [30]. CpGs have also been successfully employed as adjuvants for prophylactic vaccinations, against influenza [22, 31] and hepatitis B viruses [49].

Unmethylated CpG motifs that occur in oligonucleotides or longer DNA molecules bind to TLR9, thus, activating intracellular signal pathways that lead to the secretion of cytokines and chemokines, and upregulation of the expression of cell-surface receptors [50]. The CpG-ODN-induced innate immune response boosts vaccine-specific responses and provides protection against a wide range of microorganisms. CpG-ODNs are also among the immunostimulatory sequences (ISS) that are capable of inducing the adaptive immune system to respond to the presence of malignant cells and they can also intervene in the regulation of inflammatory responses [51].

The CpG sequence motifs used as adjuvants normally consists of six bases, with the central CG sequence being flanked on either side by two outer bases.

Three types of CpG-ODN, termed A, B and C, have been described [21, 38]:

- In type A CpG-ODNs, the CpG motifs form part of a palindromic sequence, which is bracketed by guanosines. Palindromes are sequences found within the same strand that can pair with one another via complementary bases. The nucleotides are typically linked by phosphodiester bonds and can therefore be degraded rapidly by DNases in vivo.
- Type B CpG-ODNs contain one or more CpG dinucleotides which are covalently linked exclusively by phosphorothioate bridges.
- Type C CpG-ODNs combine the features of A and B. They contain phosphorothioate bonds, as well as CpG motifs within palindromic sequences that are held together by phosphodiester linkages.

The various types of CpG-ODNs differ in their patterns of activation. Type A primarily induces the synthesis of α‑interferon (IFN-α) in pDCs, while type B is a strong activator of B lymphocytes and triggers virtually no IFN-α response. Type C possesses both of these properties to some degree.

In humans, TLR9 is found primarily in the endosomes of plasmacytoid DCs and B lymphocytes. Stimulation of TLR9 results in the production and secretion of diverse Th1-targeting cytokines and chemokines and induces an IgG class switch in B cells [52]. TLR9 is also expressed in nasal epithelia, for example in plasmacytoid dendritic cells, B cells, and in mast cells and neutrophils found in the nasal secretions. The receptor is also present on leukocytes in the bone marrow and peripheral blood [53], as well as in keratinocytes [54].

Based on the available experimental data, CpGs can be postulated to have several effects on the immune systems of allergy patients [38]. They can induce

- a local Th1-mediated immune response which is associated with suppression of the allergic reaction driven by the Th2 response,
- production of IFN-α, which has the potential to convert the Th2 response into a Th1/Treg response in human PBMCs in vitro [55],
- down-regulation of IgE synthesis, and
- a reduction in the activities of effector cells such as mast cells, basophils and eosinophils as a consequence of the stimulation of TLR9 [53].

## Arguments for the use of VLPs as immunotherapeutic agents

Over the course of evolution, the human immune system has developed highly effective mechanisms that enable it to recognize and respond to highly repetitive molecular structures which are characteristic of pathogenic microorganisms such as viruses and bacteria. This is one reason why viruses and viral proteins serve as strong activators of the human immune system. VLPs mimic the repetitive character of the structure of virus coats, and they form spontaneously during the synthesis of specific viral capsid proteins. However, unlike true viruses, VLPs do not carry a viral genome and hence are not infectious. Normally they are very well tolerated and highly immunogenic [21, 56].

Antigenic determinants displayed on the surface of VLPs are recognized by B cell receptors, and the particles themselves are efficiently taken up by antigen-presenting cells. Following intracellular processing, T cell epitopes present on the VLP coats are transported to the plasma membrane of the APC in association with MHC molecules and presented to interacting immune cells.

One other property of VLPs makes them particularly suitable for use in allergy sufferers: Allergens that have been coupled to VLPs are unable to provoke anaphylactic reactions in allergenic individuals. This feature has been demonstrated experimentally for several VLP platforms recently [23, 57, 58] and is a consequence of the physicochemical differences between free allergens and allergens present on the surfaces of VLPs [59].

VLPs can play a dual role in allergy vaccination. First, they act as an adjuvant to facilitate antigen presentation and, second, they can help to dampen the Th2 response by enhancing Th1 polarization, which is important for sustained immunity to the allergen.

## VLPs and CpG motifs

VLPs are supramolecular particles with a diameter of between 25 and 100 nm [56]. A key characteristic of the constituents of VLPs is their ability to spontaneously assemble into virus-like particles. In some VLPs, such as those based on the coat protein of bacteriophage Qbeta, this assembly process occurs immediately following the addition of fragments of RNA or DNA. Thus nucleic acids can be self-catalytically packed into these VLPs. This has been shown for QbG10, a Qbeta-based VLP that contains oligonucleotides bearing CpG motifs of type A [21, 56].

VLPs possess essentially the same key immunological features as real viruses and are just as immunogenic as pathogenic viruses without inducing any of the latter’s deleterious effects. This confers several important advantages on VLPs as immunotherapeutic agents.

- The lack of genetic information that would enable them to replicate and integrate into the genome of host cells increases the safety.
- They can be produced economically under standard GMP (Good Manufacturing Procedures) conditions.
- Owing to their form and size, they are quickly drained from the lymph nodes and can induce strong humoral immune responses.
- VLPs can transport and display virtually any antigen on their surfaces. Such antigens can be produced either by genetic fusion with the VLP coat protein (as chimeras) or by chemically attaching them to the surface of the particles (as coupled epitopes).
- Immunostimulatory agents can be packaged inside VLPs.
- Depot-forming adjuvants can be included to mimic the kinetics of natural viral infections [21, 22, 60].

Over the past several years, CpG-containing oligonucleotides have been successfully employed to treat a range of clinically important conditions [51, 61–66]. Type B CpG oligonucleotides have been used as effective adjuvants in vaccines directed against hepatitis B and influenza viruses [31, 67, 68]. Furthermore, type A and type B CpGs have been deployed as adjuvants to enhance the T cell response to a variety of tumors, including malignant lymphomas [69], melanomas [70], basal cell carcinomas [71], and glioblastomas [72]. Type C CpG oligonucleotides have also been tested in a phase Ib study for use in the treatment of hepatitis C [73].

## Clinical studies of the efficacy of VLPs and TLR ligands in the treatment of allergic rhinitis

In the following, we discuss the results of four phase I clinical studies designed to assess the impact of VLPs and TLR ligands on allergic rhinitis.

Kündig et al. conducted a randomized phase I study using RNA-loaded Qβ-VLPs coupled to a synthetic 16-amino-acid fragment of the house dust mite (HDM) allergen Der p 1 (Qβ-Der p 1). Like CpG motifs, RNA is recognized by TLRs, albeit by TLR7/8, not TLR9. The agent was administered in three doses to a group of 24 healthy subjects, and the antibody titer was monitored over the course of the following 18 months [74]. The only side-effects noted were mild skin reactions at the site of injection. A rapid IgM and IgG response (in particular IgG1 and IgG3) was induced, which was specific for the Der p 1 peptide and the cognate VLPs. No increase in IgE titers could be detected. No differences in response were observed between subjects immunized by the intramuscular or subcutaneous route [74]. This study demonstrated that the VLPs were indeed capable of inducing a significant humoral immune response and showed that the immunogen was safe and was well tolerated.

In another study, Creticos et al. tested a conjugate comprising the Amb a1 antigen linked to CpG-ODN on 25 patients who were allergic to ragweed (*Ambrosia*) [75]. In this randomized, double-blind, placebo-controlled phase II study, the adult study group was injected with six times at weekly intervals. However, the primary endpoint chosen (degree of vascular permeability in nasal epithelia, as determined by the level of albumin detected in the nasal lavage) was not significantly altered by the intervention. Nevertheless, the symptoms of allergic rhinitis were alleviated and quality-of-life (QoL) scores were markedly improved during the first ragweed pollen season following treatment. Furthermore, this improvement was maintained through the following pollen season (2 years after vaccination). In agreement with these observations, levels of the allergen-specific IgE antibodies during the ragweed season were measurably reduced relative to pretreatment values No serious side effects were observed in connection with the therapy.

Senti et al. assessed the safety, tolerability and clinical efficacy of an intervention based on the use of type A CpGs delivered by Qβ-VLPs (QbG10) [76]. The vaccine was administered together with a standard house dust mite extract. The 20 patients in this open monocentric study were allergic to house dust mites. The immunotherapy was initiated by injecting subjects with progressively increasing doses of the mite extract alone, according to a standard protocol and following a cluster scheme. This was followed by injections of a mixture containing the allergen and QbG10, which were administered six times at intervals of 1–2 weeks. The clinical endpoints in this study included a conjunctival provocation test, an assessment of asthma and allergic rhinitis-related symptoms, estimates of QoL, skin prick tests, and measurement of antibody titers. The results indicated that the combination of the mite allergen with QbG10 was safe and well tolerated. No untoward reactions were observed that could be linked to the medication. The symptoms of allergic rhinitis and allergic asthmas were significantly reduced relative to pretreatment levels, and the allergen-specific IgG response clearly increased. After showing a slight initial increase, levels of the cognate IgE antibodies dropped. The authors suggested that the CpGs alone could have alleviated the allergic symptoms by activating a Th1 immune response [76]. This hypothesis is compatible with the “hygiene hypothesis”, which postulates that atopic disorders are associated with reduced exposure to microorganisms in childhood [77]. This idea is supported by epidemiological studies, which have shown that the incidence of asthma is lower in children who grow up on farms, in children with several older siblings and in children who attended daycare centers from an early age [78–80]. The positive effects of early exposure to environmental microorganisms are most probably attributable to the induction of an allergy-protective Th1 immune response, and perhaps even to a shift from a Th2- to a Th1-type response. According to this theory, it should be possible to use VLPs loaded with CpGs to create an infection-like environment in which the immune system is more likely to activate a Th1 response [24, 81].

Klimek et al. have published the results of a randomized, double-blind, multicentric, placebo-controlled phase II study, in which they made use of the QbG10 VLPs loaded with type A CpG (CYT003-QbG10) that were employed in the trial conducted by Senti et al. [37]. However, unlike Senti et al., these authors chose not to include the allergen or any of its components in the vaccine. In all, a total of 299 patients with an allergy against HDMs were enrolled and received six weekly injections of the test substance or a placebo. The primary study lasted for 9 weeks, and the clinical endpoints consisted of assessments of symptoms, medication use and QoL, as well as a conjunctival provocation test. The treatment was rated as being safe and was generally well tolerated. The symptom and medication-use scores improved significantly following the intervention in the group that had received high doses of the VLP preparation (the verum group). The authors concluded that a monotherapy with VLPs loaded with CpGs can have a positive impact on the symptoms of allergic rhinitis, even when the allergen is not administered with the VLPs. A similar study on patients suffering from allergic asthma confirmed the therapeutic potential of CYT003-QbG10 [82].

**Fig. 3:**
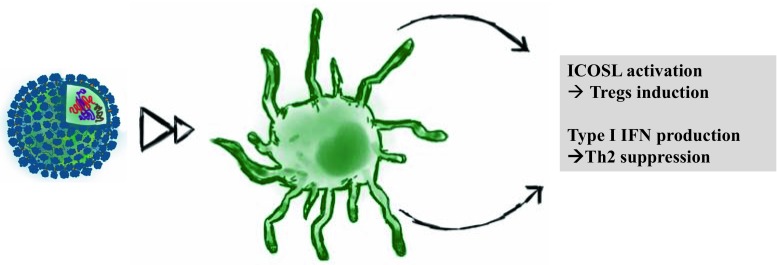
Mechanisms underlying the immunomodulatory action of CpG-loaded VLPs. *Treg* regulatory T cell; *ICOSL* inducible T cell costimulator ligand; *IFN* interferon; *Th2* T-helper cell type 2; *VLPs* virus-like particles

The mechanism responsible for the immunological effect observed here is, however, not yet understood, and further investigations will be necessary to confirm the results of this proof-of-concept study. Based on the results of in vitro studies and various experiments in animals, it is thought that the induction of a potent Th1 immune response and the production of IFN-α described above blunts the Th2 response, which is overactive in allergy sufferers (Fig. 3). Furthermore, there may a direct effect on mast cells, since they also express TLR9 [83–85]. In addition, CpGs may modify the allergic immune response by modulating the activity of the enzyme indoleamine 2,3-dioxygenase (IDO), which plays a role in T cell suppression [86]. Upregulation of IDO in specific DCs via type I interferon receptors upon stimulation with CpGs has been shown to induce T cell suppression [87]. This mechanism could in part account for the inflammation-inhibiting effects of CpGs seen in patients with allergic disorders. As mentioned above, the notion that CpG motifs could be used to treat allergies was inspired by the hygiene hypothesis. Moreover, Treg cells probably also play a prominent role in this context [86, 88]. Interestingly, CpGs have been reported to be capable of activating the expression of ICOS ligands on pDCs, which are important costimulatory molecules for the induction of regulatory T cells [89] (see Fig. 3). Since VLPs loaded with CpGs induce a strong Th1 answer, close attention will be paid to the tolerability of the treatment especially to long-term tolerability.

The significance of the VLP-specific B cell response for the positive clinical effects is still unclear. In mouse models, as well as in human subjects, VLPs can induce a B cell response in the absence of an additional adjuvant [90, 91]. In contrast, potent T cell responses can apparently be further enhanced by appropriate adjuvants [30]. Klimek et al. have shown that VLPs loaded with CpG motifs can alleviate allergic symptoms without accompanying alterations in the relevant IgE titers [37]. Several groups have shown that successful SCIT is associated with a rise in allergen-specific IgG4 and IgG1 responses [92–94]. Whether IgG2 or IgG3 antibodies make any contribution to the clinical effect is not yet known. The interaction between VLPs and antigen-presenting and regulatory T cells also needs further investigation. A better understanding of this interaction could help to answer the question of whether administration of the allergen in combination with VLPs might improve clinical outcomes. In the future, VLPs might also have a part to play in the prophylactic immunization of individuals who are at risk of developing allergies.

Other VLP platforms make use of effectively universal T cell epitopes instead of CpGs. These novel constructs are now being developed for use against diseases such as Alzheimer’s and psoriasis, but also to treat persons who are allergic to cats [23].

A vaccine designed for the treatment of peanut allergy is now undergoing preclinical development (personal communication, Bencard, Munich/Allergy Therapeutics plc, Worthing, UK).

## Conclusions

VLPs, in general and in particular, combined with CpGs as adjuvants have been successfully employed for the treatment of infectious diseases and malignancies. Thanks to the immunomodulatory characteristics associated with their viral structure, they have also been used as therapeutic agents against allergic disorders—especially for treatment of allergic rhinitis and asthma. Indeed, VLPs loaded with CpGs appear to promote the reconstitution of a physiological immune response in allergy patients, although the underlying immunological mechanisms are not yet fully understood.

Early clinical trials with various forms of VLP- and/or CpG-based vaccines have not only demonstrated that these agents are safe, but they have also been shown to have promising immunological and clinical effects (in terms of symptoms, reduction of drug use, and response to allergen challenge). Large-scale controlled studies will, however, be necessary in order to learn more about the broader clinical properties of this new technology. Nevertheless, theoretically, the use of VLPs in combination with CpG motifs has great potential in the treatment of allergic patients—but also as a prophylactic measure in individuals who are at risk of developing allergies.

## Abbreviations

APC: Antigen-presenting cells
AR: Allergic rhinitis
ARIA: Allergic Rhinitis and its Impact on Asthma (Initiative)
CpG-ODN: CpG-oligodeoxynucleotides (C, cytosine; G, guanine)
DC: Dendritic cells
FOXP3: Forkhead box protein 3
GMP: Good Manufacturing Procedures
ICOSL: Inducible costimulator ligand
IgE: Immunoglobulin E
IgG: Immunoglobulin G
IgM: Immunoglobulin M
IL: Interleukin
ISS: Immunostimulatory sequence
MHC: Major histocompatibility complex
QoL: Quality of life
SCIT: Subcutaneous immunotherapy
SIT: Specific immunotherapy
T-bet: T-box expressed in T cells
TGF: Transforming growth factor
Th: T-helper cell
TLR: Toll-like receptor
TLT: Toll-like receptor family
Treg: Regulatory T cells
VLP: Virus-like particle
